# Encapsulation of primary dopaminergic neurons in a GDNF-loaded collagen hydrogel increases their survival, re-innervation and function after intra-striatal transplantation

**DOI:** 10.1038/s41598-017-15970-w

**Published:** 2017-11-22

**Authors:** Niamh Moriarty, Abhay Pandit, Eilís Dowd

**Affiliations:** 1 Pharmacology & Therapeutics, National University of Ireland, Galway, Ireland; 2 CÚRAM Centre for Research in Medical Devices, National University of Ireland, Galway, Ireland

## Abstract

Poor graft survival limits the use of primary dopaminergic neurons for neural repair in Parkinson’s disease. Injectable hydrogels have the potential to significantly improve the outcome of such reparative approaches by providing a physical matrix for cell encapsulation which can be further enriched with pro-survival factors. Therefore, this study sought to determine the survival and efficacy of primary dopaminergic grafts after intra-striatal delivery in a glial-derived neurotrophic factor (GDNF)-loaded collagen hydrogel in a rat model of Parkinson’s disease. After intra-striatal transplantation into the lesioned striatum, the GDNF-enriched collagen hydrogel significantly improved the survival of dopaminergic neurons in the graft (5-fold), increased their capacity for striatal re-innervation (3-fold), and enhanced their functional efficacy. Additional studies suggested that this was due to the hydrogel’s ability to retain GDNF in the microenvironment of the graft, and to protect the transplanted cells from the host immune response. In conclusion, the encapsulation of dopaminergic neurons in a GDNF-loaded hydrogel dramatically increased their survival and function, providing further evidence of the potential of biomaterials for neural transplantation and brain repair in neurodegenerative diseases such as Parkinson’s disease.

## Introduction

The relatively selective loss of dopaminergic neurons from the substantia nigra *pars compacta* makes Parkinson’s disease an ideal candidate for cell replacement therapies^1,2^. To date, the focus of cell therapies in Parkinson’s disease has been on the transplantation of dopamine neuron-rich foetal ventral mesencephalon (VM) grafts which have shown to both survive and re-innervate the striatum post-transplantation, whilst also restoring motor function^3–7^. However, despite long-term symptomatic relief in some patients, significant limitations, including poor survival post-transplantation, prevent this therapy being utilised as a potential restorative approach for Parkinson’s disease^8^. VM grafts contain diverse cell populations, the least abundant of which is dopaminergic neurons, and less than 20% of these neurons survive transplantation^9^. Thus, poor survival, the sheer volume of human foetal tissue required (10 per grafted hemisphere), and the associated ethical concerns has highlighted an urgent need for improved methodologies to enhance dopamine neuron survival rates post-transplantation.

While the efficacy of dopamine neuron-rich foetal VM grafts is still being investigated clinically through the TRANSEURO consortium^10^, the field of cell replacement therapy in Parkinson’s disease is moving towards more readily available dopaminergic cell sources, such as those derived from embryonic stem cells and induced pluripotent stem cells^11^. While these cells show extrordinary regenerative potential, their use is still in the experimental stages and has not yet reached a clinical setting. With this is mind, dopamine neuron-rich foetal VM grafts are an extremely well established cell type and are therefore optimal for testing the potential of biomaterial scaffolds to improve the survival and efficacy of such cell regenerative therapies.

The majority of cell death in VM grafts occurs through apoptosis at various points of the transplantation process^12^ by factors such as detachment from the extracellular matrix during tissue dissection^13^, growth factor deprivation upon transplantation^14^, and recruitment of host neuro-immune cells to the exogenous graft^15^. Each of these stages provides a target point of intervention at which graft survival could be improved. Injectable scaffolds, such as *in situ* forming hydrogels, may provide a delivery platform to improve grafted cell survival after transplantation. These hydrogels could potentially increase cell engraftment by providing a supportive environment for cell adhesion, creating a physical barrier between the transplanted cells and the host neuro-immune cells and by providing a reservoir for localised growth factor delivery^16^. A particular scaffold of interest, collagen, is a clinically accepted, highly abundant and natural extracellular matrix that is used for a variety of applications^17–24^. The injectable nature of collagen hydrogels, coupled with their ability to support and immunoisolate cells, whilst simultaneously delivering trophic factors in a localised manner, creates a natural scaffold with the potential to improve the transplantation of dopaminergic neurons. Despite this, the intra-cerebral use of collagen hydrogels has not been well established as a delivery platform in its own right.

Thus, this study aimed to assess the use of a glial-derived neurotrophic factor (GDNF)-loaded collagen hydrogel for the transplantation of primary dopaminergic neurons to the Parkinsonian brain. GDNF was selected as the growth factor in this study as it is well established as a neurotrophin for developing dopaminergic neurons^25^. We hypothesised that the type 1 collagen hydrogel would provide a local GDNF reservoir and reduce the host immune response to the transplanted cells, thereby improving the overall survival, re-innervation and functionality of primary dopaminergic neurons after intra-striatal transplantation.

## Methods

### *In vitro*/*ex vivo* experimental design

Before undertaking *in vivo* studies, *in vitro* and *ex vivo* studies were completed in order to determine the cytocompatibility of the collagen hydrogels. This was assessed using alamarBlue® cell viability assay and immunocytochemistry on bone marrow-derived mesenchymal stem cells (MSC) and/or primary embryonic day 14 (E14) VM cell cultures. Subsequently a series of *in vivo* studies to optimise the collagen hydrogel for VM cell transplantation were conducted.

### *In vivo* experimental designs

#### Preliminary in vivo assessment of the impact of hydrogel cross-linking on grafted cell viability

As the concentration of poly(ethylene glycol) ether tetrasuccinimidyl glutarate (4s-StarPEG) used to crosslink collagen will affect the intensity of gelation, it was important to determine to what extent the hydrogel can be cross-linked without negatively impacting graft survival. The final level of cross-linking will be chosen based on successful graft survival. In order to determine the optimum level of hydrogel cross-linking for cell delivery, an *in vivo* pilot study using male Sprague-Dawley rats (n = 24) was carried out. Rats were divided into two groups to receive either a bilateral intra-striatal transplant of green fluorescent protein (GFP)-MSCs (30,000 cells/3 µl) delivered in transplantation media or encapsulated in a collagen hydrogel of various 4s-StarPEG concentrations (1, 2, or 4 mg/ml). The animals were then sacrificed for *post mortem* analysis at days 1, 4 and 7 post transplantation (n = 4 per group, per time point). GFP-MSCs were used as they are easily detected post-transplantation and are ideal to assess whether the intensity of gelation is suitable for graft survival. A schematic of this experimental design is shown in the results section.

#### Preliminary in vivo assessment of the impact of the collagen hydrogel on encapsulated VM cells

Once the optimal level of hydrogel cross-linking was determined, an *in vivo* pilot study was carried out to assess if the collagen hydrogel will impact the striatal re-innervation from encapsulated VM cells. Male Sprague-Dawley rats (n = 24) received a unilateral intra-medial forebrain bundle (MFB) 6-hydroxydopamine (6-OHDA) lesion (3 µl). Two weeks later, all rats were subjected to methamphethamine-induced rotations. Based on these results, rats were then performance matched into six groups (n = 4 per group) to receive intra-striatal transplants of E14 VM cells of various densities (200,000, 300,000 or 400,000 cells per 6 µl) in transplantation media or encapsulated in collagen hydrogel (cross-linked with 4 mg/ml 4s-StarPEG). The animals were then sacrificed two-weeks post-transplantation for *post mortem* assessment. A schematic of this experimental design is shown in the results section.

#### Preliminary in vivo assessment of GDNF retention within the collagen hydrogel

To assess the striatal retention of human recombinant GDNF when encapsulated within the collagen hydrogel, male Sprague-Dawley rats (n = 12) received bilateral intra-striatal infusions of GDNF (1000 ng) as either a bolus or encapsulated in a collagen hydrogel (6 µl per rat). The animals were then sacrificed for *post mortem* assessment at days 1, 2 and 4 post transplantation (n = 4 per group, per time point). A schematic of this experimental design is shown in the results section.

#### Main in vivo study to assess the long-term survival, reinnervation and functionality of grafted VM cells encapsulated in a GDNF-loaded collagen hydrogel

**Figure 1:**
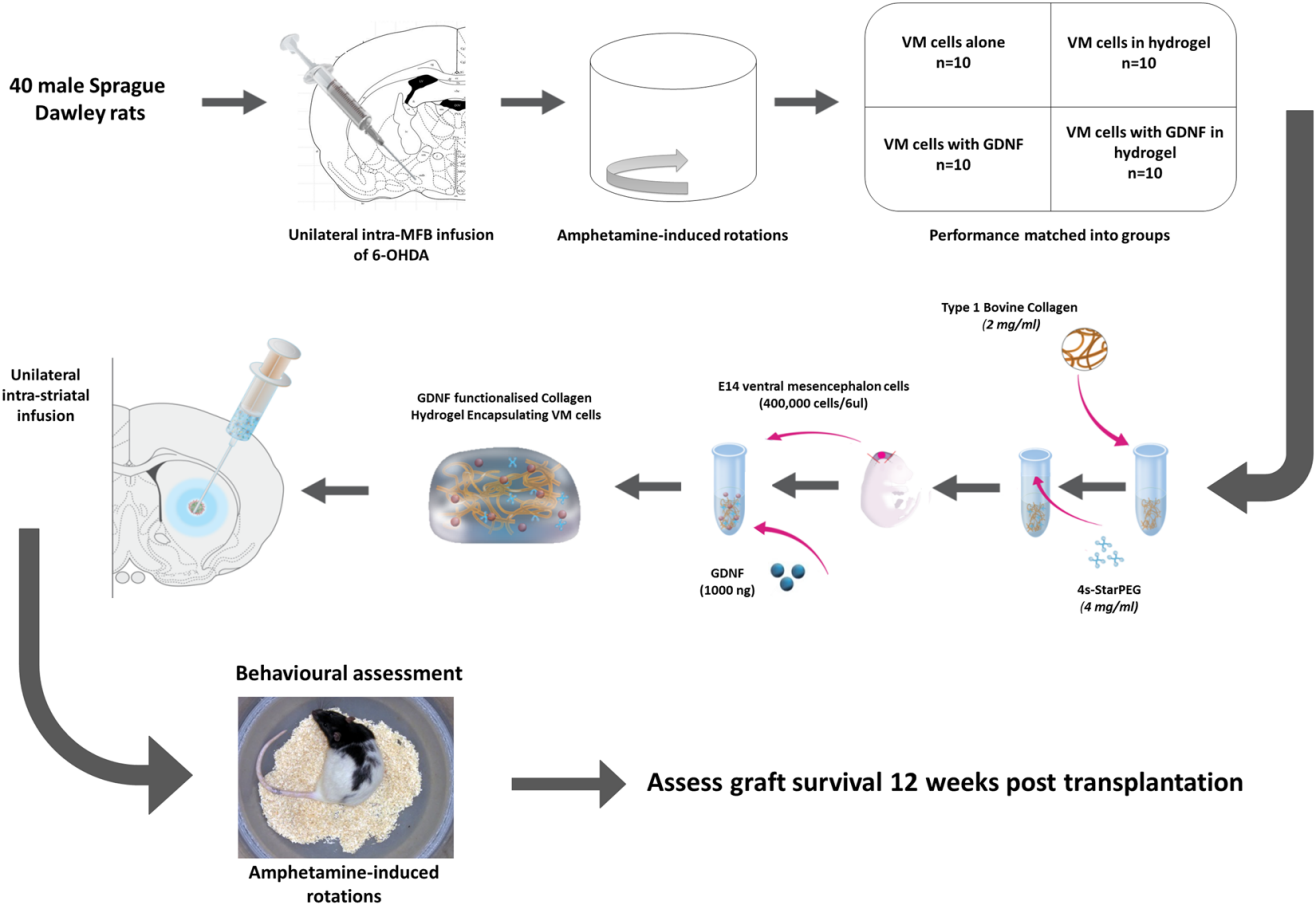
Main *in vivo* experimental design. Primary dopaminergic neurons (400,000 cells) dissected from the VM of E14 foetuses were encapsulated in a GDNF-loaded (1000 ng) type 1 bovine collagen hydrogel (2 mg/ml), cross-linked with 4s-StarPEG (4 mg/ml) and kept on ice prior to unilateral transplantation into the 6-OHDA MFB-lesioned striatum. After 12 weeks of behavioural assessment, the adult rat brains were assessed for host response, collagen degradation, GDNF release, host response, cell survival and striatal re-innervation.

After the initial *in vitro, ex vivo* and *in vivo* analyses, the main *in vivo* study was conducted to assess the long term survival and efficacy of E14 VM cells encapsulated in a GDNF-loaded collagen hydrogel. Male Sprague-Dawley rats (n = 40) received a unilateral intra-MFB 6-OHDA lesion (3 µl). Two weeks later, rats underwent post-lesion methamphetamine-induced rotations. Based on these results, rats were performance matched into four groups to receive intra-striatal transplants of VM cells alone (400,000 per 6 µl), VM cells with GDNF (1000 ng), VM cells encapsulated in a collagen hydrogel (cross-linked with 4 mg/ml 4s-StarPEG) or VM cells encapsulated in a GDNF-loaded collagen hydrogel. Methamphetamine-induced rotations resumed three weeks post-transplantation and were carried out at three week intervals for a total of twelve weeks. The animals were then sacrificed for *post mortem* assessment. A schematic of this experimental design is shown in Fig. 1.

### Animals

All experiments involving the use of animals for procedures and cell preparations were carried out in accordance with relevant guidelines and regulations, were completed under licence by the Irish Department of Health and Children and the Irish Health Products Regulatory Authority, were performed in compliance with the European Union Directive 2010/63/EU and S.I No. 543 of 2012, and were approved by the Animal Care and Research Ethics Committee at the National University of Ireland, Galway. Male Sprague-Dawley rats (weighing 200–225 g on arrival) and time-mated female Sprague-Dawley rats were sourced from Charles River, UK. Animals were housed in groups of four per cage, on a 12:12 h light/dark cycle, at 19–23 °C, with relative humidity levels maintained between 40 and 70%. For the duration of the experiment, animals were allowed food and water *ad libitum*. All behavioural testing and *ex vivo* analyses were carried out by an experimenter who was blind to the treatment of the animals.

### Cell culture

For E14 VM cultures, E14 embryos were obtained by laparotomy from time-mated female Sprague-Dawley rats following quick decapitation under isoflorane (5% in 0.5 L 0_2_). The VM was micro-dissected from each embryo as previously described^26^. Dissected VM tissue was centrifuged at 1100 rpm for 5 min at 4 °C. The tissue pellet was incubated in 40% trypsin-Hank’s balanced salt solution (HBSS) for 4 min, at 37 °C with 5% CO_2_. Foetal calf serum (FCS) was then added to the tissue and centrifuged at 1100 rpm for 5 min at 4 °C. The cell pellet was then resuspended in 1 ml of plating media (Dulbecco’s modified Eagle’s medium/F12, 0.6% D-glucose, 1% L-glutamine, 1% FCS and 2% B27), first using a P1000 Gilson pipette, followed by a 25 gauge needle and syringe. Cell density was estimated using a haemocytometer. For *in vitro* studies cells were resuspended at 2000 cells/µl and for *in vivo* studies cells were resuspended at 166,666 cells/µl.

Bone marrow-derived MSCs were extracted from the femora and tibiae of GFP transgenic Sprague-Dawley rats and characterized as MSCs as described previously^27^. MSCs were then cultured in 1:1 Dulbecco’s modified Eagle’s medium: alpha-minimum essential media (DMEM:Alpha-MEM) mix containing 10% FCS and 1% penicillin/streptomycin at 37 °C with 5% CO_2_.

### Fabrication of cross-linked type 1 bovine collagen hydrogels

During the preparation of collagen hydrogels, all components were maintained on ice to prevent premature gelation. For a final volume of 100 µl, 40 µl of 5 mg/ml type 1 collagen (Vornia Biomaterials), neutralised with 1 M NaOH until PH 7 reached, was added to 20 µl of 10x phosphate buffer saline (PBS) containing 4s-StarPEG. 40 µl of transplantation media (un-seeded hydrogels), cell suspension (seeded hydrogels) or human recombinant GDNF enriched cell suspension (GDNF-loaded hydrogels) was then added to the collagen/PBS/cross-linker solution and mixed thoroughly. For *in vitro* experiments, 50 µl samples were transferred to a previously sterilised (UV radiation) super hydrophobic surface (Teflon^®^) and placed at 37 °C to gel. For *in vivo* experiments, the cell-seeded collagen hydrogel was maintained on ice prior to transplantation to prevent premature gelation.

### Cell viability assays

In order to determine the effect of the collagen hydrogels on MSC viability, MSCs were seeded at a density of 20,000 cells per well of a 24 well-plate and left overnight to attach. MSCs were then either incubated with unseeded collagen hydrogels (2 × 50 µl gels per well) of various 4s-StarPEG concentrations (1, 2, or 4 mg/ml) for 48 h or left untreated. As an indicative measure of cell viability, metabolic activity of the cells was assessed using the alamarBlue^®^ assay as previously described^28^. Briefly, 100 µl of a 10% solution of alamarBlue^®^ (Invitrogen) in HBSS was added to each well and incubated for 3 h. Absorbance was read at 550 nm and 595 nm using a Varioskan Flash plate reader (Thermo Scientific) with SkanIt^®^ software. MSC viability was assessed by normalisation of all results to controls.

To determine the effect of collagen hydrogels on VM cell viability, E14 VM cells were seeded on poly-D-lysine (Sigma) coated 24 well plates, at a density of 100,000 cells per well in 500 µl of plating media at 37 °C with 5% CO_2_ for 48 h. As above, the E14 VM cells were either incubated with unseeded collagen hydrogels (2 × 50 µl gels per well) of various 4s-StarPEG concentrations (1, 2, or 4 mg/ml) for 48 h or left untreated. Once again, metabolic activity was assessed using the alamarBlue^®^ assay as described above.

### E14 VM cell immunocytochemistry

Dopaminergic neuron survival and outgrowth was assessed 48 h after treatment with collagen hydrogels (as described above) using tyrosine hydroxylase (TH) and beta-III tubulin immunocytochemistry, respectively. VM cultures were fixed with 4% paraformaldehyde (PFA) for 30 min, followed by three washes in tris-buffered saline (TBS) with 0.2% triton-X-100 for permeabilization. Cultures were then incubated in blocking serum (5% bovine serum albumin in TBS with 0.2% triton-X-100) for 1 h at room temperature, before being subsequently incubated with primary antibody (Mouse anti-TH, 1:1000, Millipore; Mouse anti-beta III tubulin, 1:200, Millipore) diluted with 1% bovine serum albumin in TBS with 0.2% triton-X-100 at room temperature overnight. Following 3 × 10 min washes with TBS, cultures were incubated in rabbit anti-mouse AF 488 conjugated secondary antibodies (1:1000, Biosciences) in 1% bovine serum albumin in TBS, at room temperature for 3 h in darkness. Cultures were then counterstained with 4′,6-diamidino-2-phenylindole (DAPI) (1 µg/ml in TBS, Sigma) for 5 min. Following 3 × 10 min washes in TBS, cultures were stored in 0.1% TBS azide at 4 °C until imaging. Negative controls, where no primary antibody was added were also prepared (data not shown).

### 6-OHDA lesions, transplantation and rotational behaviour

All surgeries were performed under isoflurane anaesthesia (5% in O_2_ for induction and 2% in O_2_ for maintenance) in a stereotaxic frame with the nose bar set at −4.5 (intra-MFB) or −2.3 (intra-striatal). The striatum was infused unilaterally or bilaterally at coordinates Anterior-Posterior (AP) = 0.0, Medial-Lateral (ML) ±3.7 (from bregma) and Dorsal-Ventral (DV) −5.0 below dura, while the MFB was infused unilaterally at coordinates AP −4.0, ML −1.3 (from bregma) and DV −7.0 below dura. Infusions were completed at a total volume of either 3 µl (6-OHDA lesion and MSC transplants) or 6 µl (VM transplants and collagen hydrogels) at a rate of 1 µl/min with a further 2 min allowed for diffusion. Dopaminergic asymmetry in lesioned and transplanted rats was assessed via rotational behaviour using the dopaminergic stimulant methamphetamine (5 mg/ml i.p.) as previously described^29,30^.

### Immunohistochemistry (IHC)

Animals were sacrificed by terminal anaesthesia (50 mg/kg pentobarbital intraperitoneal (i.p)) and transcardially perfused with 100 ml heparinised saline followed by 150 ml of 4% PFA. Brains were rapidly removed and placed in 4% PFA overnight before being cryoprotected in 25% sucrose solution. Serial coronal sections (30 µm) were cut using a freezing stage sledge microtome (Bright, Cambridgeshire, UK) and free floating IHC for TH, collagen, GDNF, microglial activation (cd11b) and astrocyte recruitment (glial fibrillary acidic protein (GFAP)) was performed as previously described^29,31^. In short, endogenous peroxidise activity was quenched using a solution of 3% hydrogen peroxidase and 10% methanol in distilled water. Non-specific binding was blocked using 3% normal horse serum (TH, GDNF and cd11b) or normal goat serum (Collagen and GFAP) in TBS with 0.2% Triton-X-100. Primary antibody (Mouse anti-TH, 1:1000, Millipore; Rabbit anti-collagen, 1:1000, Abcam; Mouse anti-GDNF, 1:200, R & D systems; Mouse anti-cd11b, 1:400, Millipore; Rabbit anti-GFAP, 1:2000, Dako) was diluted in TBS with 0.2% triton-X-100, added to sections and incubated at room temperature overnight. Sections were incubated in secondary antibody (Horse anti-mouse, 1:200, Vector; Goat anti-rabbit, 1:200, Jackson ImmunoResearch) for 3 h at room temperature. A streptavidin-biotin-horseradish peroxidise solution (Vector, UK) was subsequently added to sections and allowed to incubate for 2 h. The development of staining was carried out using a 0.5% solution of diaminobenzidine tetra hydrochloride (DAB, Sigma, Ireland) in TBS containing 0.3 µl/ml of hydrogen peroxide. Sections were mounted onto gelatin-coated slides, dehydrated in a series of accending alcohols, cleared in xylene and finally coverslipped using DPX mountant for DAB stained sections (Sigma) or ‘fluoromount’ fluorescent mounting medium for GFP-expressing sections (Sigma).

### Image analysis

*In vitro* image analysis consisted of counting the number of TH^+^ cells and the area of beta-III tubulin fluorescence. TH^+^ cells were quantified from five randomly selected sample sites per well, in three technical replicates per experimental condition, with three biological replicates. The level of beta-III tubulin florescence was quantified by measuring the threshold area of each image using ImageJ software.

Graft volume, re-innervation volume, collagen volume, GDNF volume and the volume of microgliosis/astrocytosis were assessed using ImageJ software, as described previously^31^. For MSC graft volume, the transplant was identified directly by GFP expression from fluorescent photomicrographs, whereas VM graft, collagen, GDNF, cd11b and GFAP expression were identified in DAB stained sections. For each of these experimental outcomes, volume was measured using cross-sectional areas measured on a one in six series of sections throughout the rosto-caudal axis of the striatum. All sections containing either GFP expression or elevated DAB staining were used in the analysis. The total number of transplanted VM cells was determined by counting individual TH^+^ cell bodies in the transplanted region and correcting using Abercrombie’s equation. All sections containing TH^+^ cell bodies were used.

### Statistical analysis

All data are expressed as mean ± standard error of the mean, and were analysed using 1-way, 2-way or 2-way repeated measures analysis of variance (ANOVA) as appropriate, with *post hoc* Bonferroni test when required. Throughout the results text, the main effects from the initial ANOVA are cited in the body of the results, while the results of the *post-hoc* analyses are shown on the corresponding figure and explained in the figure legend.

### Data availability

All data generated or analysed during this study are included in this published article.

## Results

### *In vitro/ex vivo* assessment of the impact of differently cross-linked collagen hydrogels on cell viability

**Figure 2:**
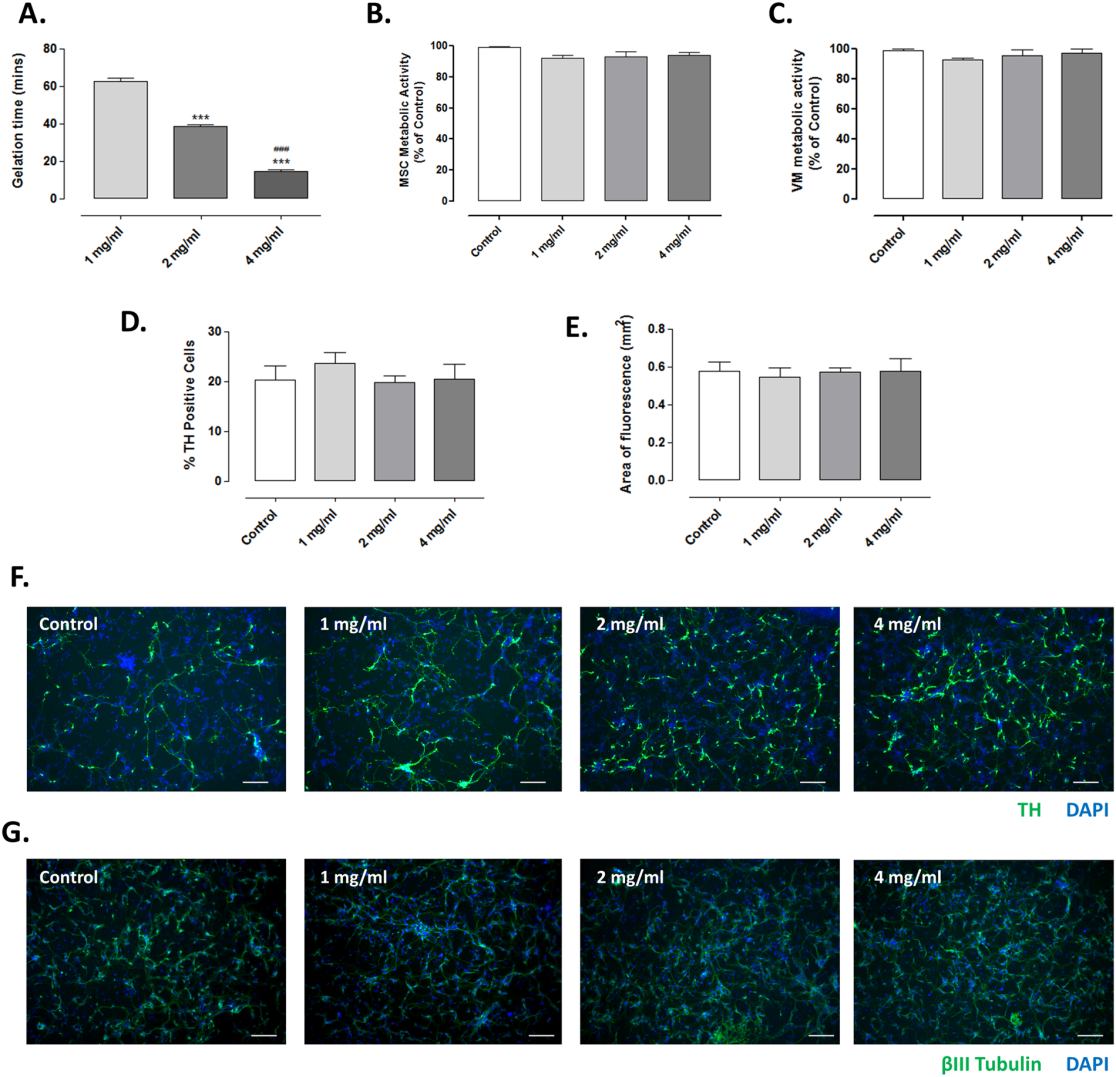
Collagen hydrogels of all cross-linker concentrations are cytocompatible *in vitro*/*ex vivo*. The cross-linking of hydrogels with rising levels of 4s-StarPEG significantly decreased the time required for gelation (**A**). The incubation of collagen hydrogels cross-linked with various 4s-StarPEG concentrations had no negative effect on the metabolic activity of (**B**) MSCs or (**C**) VM cells. Similarly, TH and β-III tubulin immunocytochemistry showed that the incubation of the hydrogels with VM cultures had no negative impact on (**D**) the number of surviving TH^+^ primary dopaminergic neurons or (**E**) the neural outgrowth from these cells, respectively. Photomicrographs (**F**,**G**) represent TH and β-III tubulin immunocytochemistry, counterstained with DAPI, respectively. Scale bar represents 100 µm. Data are represented as mean ± SEM and were analysed by one-way ANOVA with *post-hoc* Bonferroni. ^***^P < 0.001 vs. 1 mg/ml; ^###^P < 0.001 vs. 2 mg/ml.

Prior to conducting *in vivo* experiments, collagen hydrogels of different 4s-StarPEG concentrations were formulated to determine their *in vitro* cytocompatibility. It was shown that the higher the concentration of 4s-StarPEG used, the shorter the time taken for gelation (Fig. 2A; Group, *F*_(2,6)_ = 404.30, *P* < 0.0001).

In order to determine if the collagen hydrogels of rising cross-linker concentrations (1, 2 or 4 mg/ml 4s-StarPEG) had any detrimental effects on the viability of MSCs, they were incubated with pre-formed collagen hydrogels. None of the collagen hydrogels were found to have a negative impact on cell survival (Fig. 2B; Group, *F*_(2,6)_ = 0.14, *P* > 0.05).

Once it was confirmed that the differently cross-linked collagen hydrogels did not impact cell survival, we then wanted to confirm that the presence of collagen hydrogels in VM cultures did not affect survival or neural outgrowth of the TH^+^ primary dopaminergic neurons. The presence of hydrogels did not have any negative impact on overall VM cell viability (Fig. 2C; Group, *F*_(2,6)_ = 0.5757, *P* > 0.05), and when the survival of dopaminergic neurons within these cultures was assessed, the hydrogels also had no negative effect on the number of surviving TH^+^ cells (Fig. 2D; Group, *F*_(3,16)_ = 0.5143, *P* > 0.05) and importantly, the presence of the hydrogels did not hinder the neural outgrowth from these TH^+^ dopaminergic neurons (Fig. 2E; Group, *F*_(3,12)_ = 0.0865, *P* > 0.05). This indicates that increasing levels of cross-linker in hydrogels is cytocompatible with these cells, at least when they are in an *ex vivo* cell culture system.

### *In vivo* assessment of the impact of hydrogel cross-linking on grafted cell viability

**Figure 3:**
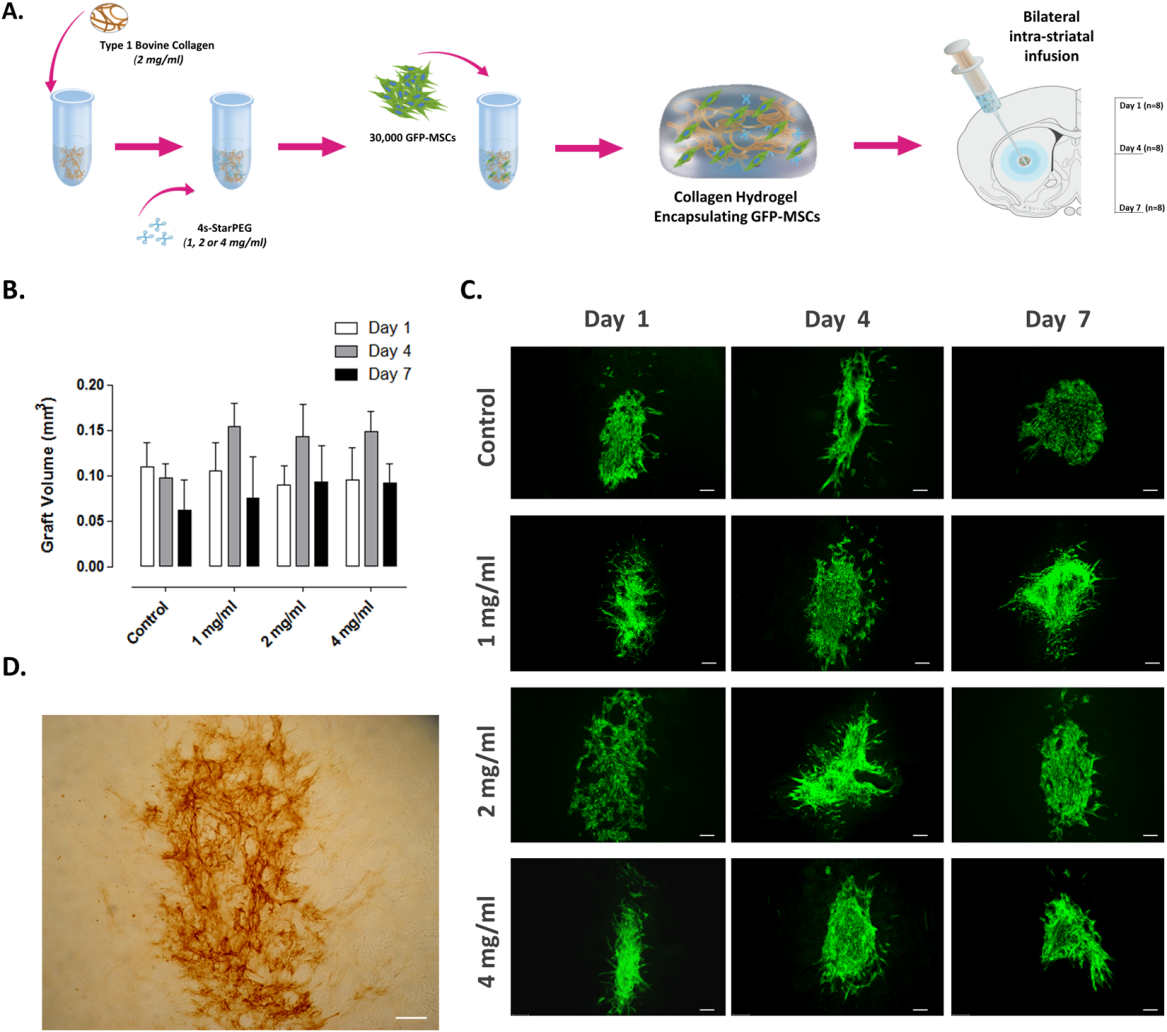
*In vivo* assessment of the impact of hydrogel cross-linking on grafted cell viability. 30,000 MSCs were delivered bilaterally to the striatum in either transplantation media or a collagen hydrogel of various 4s-StarPEG concentrations and graft volume was assessed at days 1, 4 and 7 post-transplantation (**A**). MSCs were extracted from the bone-marrow of GFP transgenic rats, meaning grafts could be easily identified using fluorescent microscopy. The encapsulation of MSCs in hydrogels of increasing levels of 4s-StarPEG had no negative effect on graft volume in each group, at each time-point (**B**,**C**). Collagen IHC confirmed the *in situ* polymerisation of hydrogels and their presence at 7 days post-transplantation (**D**). Scale bar represents 100 µm. Data are represented as mean ± SEM and were analysed by two-way ANOVA with *post-hoc* Bonferroni.

Having determined that the collagen hydrogels are cytocompatible *in vitro*, we then sought to determine the optimal level of 4s-StarPEG cross-linker for encapsulation of cells in a collagen hydrogel. To investigate this, the survival of GFP-MSCs either delivered in control transplantation media or encapsulated in collagen hydrogels cross-linked with 1, 2 or 4 mg/ml 4s-StarPEG was assessed at days 1, 4 and 7 post-transplantation. A schematic of this experimental design is shown in Fig. 3A. Since the MSCs were extracted from the bone marrow of GFP transgenic rats, the cellular grafts could be easily visualised in the striatum using florescent microscopy. MSC graft volume was similar between each group at each time-point (Fig. 3B; Group, *F*_(3,24)_ = 0.11, *P* > 0.05) indicating that the encapsulation of cells inside the collagen hydrogels, of any cross-linker concentration, did not impact graft survival. Furthermore, the collagen hydrogels successfully formed *in situ*, were still present 7 days post-infusion (Fig. 3D) and did not result in any striatal damage (not shown). This confirmed that the collagen hydrogels were suitable for intra-striatal cell delivery.

### *In vivo* assessment of the impact of the collagen hydrogel on encapsulated VM cells

**Figure 4:**
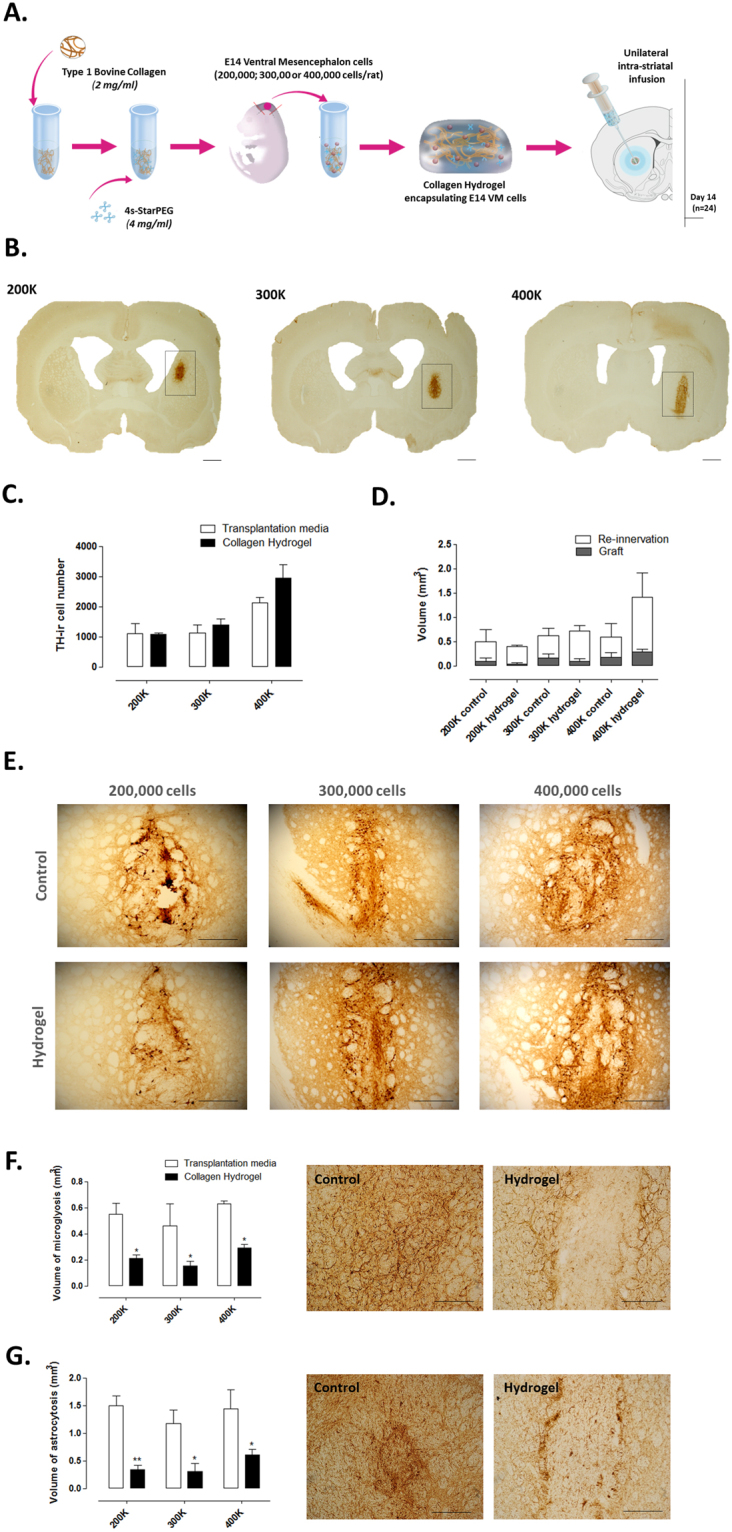
*In vivo* assessment of the impact of the collagen hydrogel on encapsulated VM cells. VM cells of various cell densities (200,000; 300,000 or 400,000 cells) were delivered unilaterally to the lesioned striatum in either transplantation media or a collagen hydrogel (cross-linked with 4 mg/ml 4s-StarPEG) and assessed for graft survival, efficacy and host-immune response 14 days post-transplantation (**A**). Collagen IHC confirmed the *in situ* formation of hydrogels and their presence at 14 days post-transplantation (**B**). The encapsulation of VM cells in a collagen hydrogel had no negative effect on the survival of primary dopaminergic neurons (**C**,**E**) or their ability to re-innervate the striatum (**D**,**E**). This indicates that the collagen hydrogel supports the *in vivo* delivery of a high density of VM cells. The host response to the transplanted graft was assessed using CD11b and GFAP IHC. The encapsulation of cells within the collagen hydrogel significantly decreased the volume of microgliosis (**F**) and astrocytosis (**G**). This indicated that the collagen hydrogel can act as a physical barrier between the transplanted cells and the host neuro-immune cells. This reduction in microgliosis and astrocytosis to the encapsulated VM cells (400,000 cells) can be seen in photomicrographs F and G, respectively. Scale bar represents 1 mm. Data is represented as mean ± SEM and were analysed by two-way ANOVA with *post-hoc* Bonferroni. ^*^ *P* < 0.05, ^**^ *P* < 0.01 vs. relative control.

Having demonstrated that the collagen hydrogels are highly cytocompatible (with MSCs) and are well tolerated *in vivo*, further studies were carried out using a collagen hydrogel cross-linked with 4 mg/ml of 4s-StarPEG. We then sought to determine the impact of the collagen hydrogel on the striatal re-innervation from different densities of VM cells encapsulated within it (to ensure that the hydrogel did not impede striatal reinnervation). A schematic of this experimental design is shown in Fig. 4A.

The optimised collagen hydrogel successfully formed *in situ* after intra-striatal delivery and was still present 14 days post-transplantation (Fig. 4B). VM cells at cell densities of 200,000; 300,000 or 400,000 cells were delivered either within control transplantation media or the collagen hydrogel. TH^+^ cell survival and striatal re-innervation was then assessed at 14 days post-transplantation. The delivery of VM cells in the collagen hydrogel did not have any significant impact on either the survival of cells (Fig. 4C,E; Group, *F*_(2,12)_ = 1.23, *P* > 0.05) or on their ability to re-innervation the striatum (Fig. 4D,E; Group, *F*_(2,12)_ = 1.183, *P* > 0.05). Thus, although the hydrogel was not successful at improving the number of surviving TH^+^ cells, it was not detrimental to graft survival and did not impede striatal re-innervation from within the hydrogel, confirming it is possible to deliver the highest density of VM cells (400,000 cells) without any negative effects on graft survival. We then sought to establish whether encapsulation of cells in a collagen hydrogel would create a physical barrier between the transplanted VM cells and host neuro-immune cells and thereby reduce the host immune response to the transplanted graft. As expected, the cells delivered in control transplantation media elicited a substantial host immune response. The delivery of cells in the collagen hydrogel significantly decreased the volume of microgliosis around the graft site (Fig. 4F; Group, *F*_(2,17)_ = 23.03, *P* < 0.05). Similarly, the delivery of cells in the collagen hydrogel significantly decreased the volume of astrocytosis around the graft site (Fig. 4G; Group, *F*_(2,18)_ = 34.01, *P* < 0.0001).

### *In vivo* assessment of GDNF retention within the collagen hydrogel

**Figure 5:**
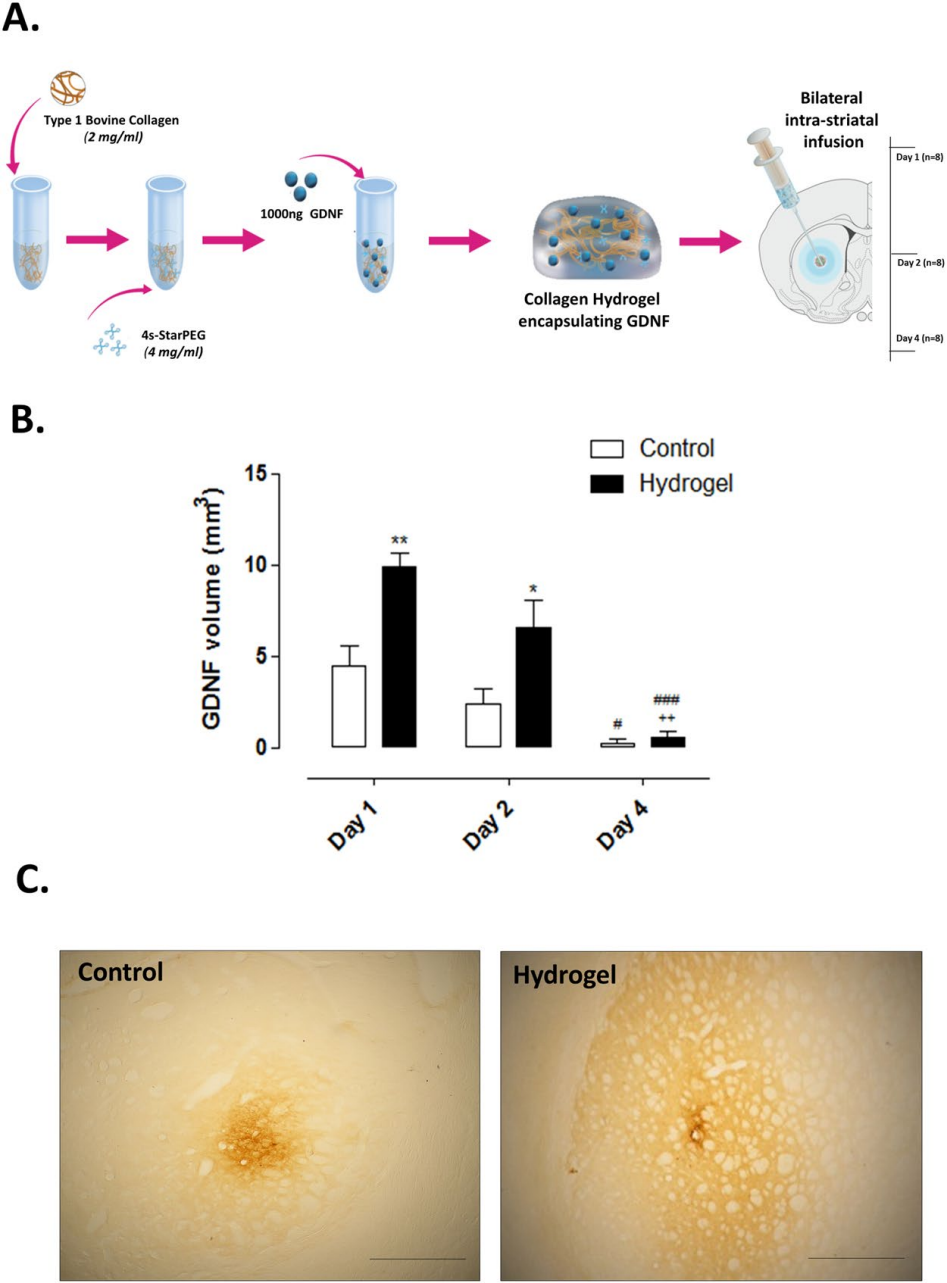
*In vivo* assessment of GDNF retention within the collagen hydrogel. Recombinant human GDNF (1000 ng) was delivered bilaterally to the striatum as a bolus or in a collagen hydrogel (cross-linked with 4 mg/ml 4s-StarPEG) and the volume of GDNF staining was analysed at days 1, 2 and 4 post-transplantation (**A**). GDNF IHC showed that despite the significant depletion of GDNF by day 4, the encapsulation of GDNF in a collagen hydrogel significantly increased the volume of striatal GDNF at days 1 and 2 post-transplantation (**B**). Photomicrographs show striatal GDNF at Day 1 (**C**). Scale bar represents 1 mm. Data is represented as mean ± SEM and were analysed by two-way ANOVA with *post-hoc* Bonferroni. ^*^ *P* < 0.05, ^**^ *P* < 0.01 vs. relevant control; ^#^ *P* < 0.05, ^###^ *P* < 0.001 vs. relevant day 1; ^++^ *P* < 0.01 vs. relevant day 2.

Having established that the collagen hydrogel is suitable for the intra-striatal delivery of VM cells, we then sought to determine the impact on GDNF retention in the surrounding striatum. Intra-striatal GDNF was delivered either as a bolus or in a collagen hydrogel and assessed at days 1, 2 and 4. A schematic of this experimental design is shown in Fig. 5A. Although the volume of striatal GDNF declined over time (Fig. 5B; Time, *F*_(2,12)_ = 25.63, *P* < 0.0001), delivery within the hydrogel significantly improved retention at the early time points (Fig. 5B,C; Group, *F*_(2,12)_ = 3.95, *P* < 0.05).

### Pivotal *in vivo* study to assess the long-term survival, re-innervation and functionality of grafted VM cells encapsulated in a GDNF-loaded collagen hydrogel

Having established that the collagen hydrogel is well tolerated *in vivo*, supports cell survival and striatal re-innervation, reduces the host immune response and retains GDNF at a higher volume in the striatum, we then wanted to investigate the effect of a GDNF-loaded collagen hydrogel on dopaminergic cell survival, striatal re-innervation and functional efficacy. A schematic of this experimental design is found in Fig. 1.

#### Impact of the GDNF-loaded collagen hydrogel on host immune response

**Figure 6:**
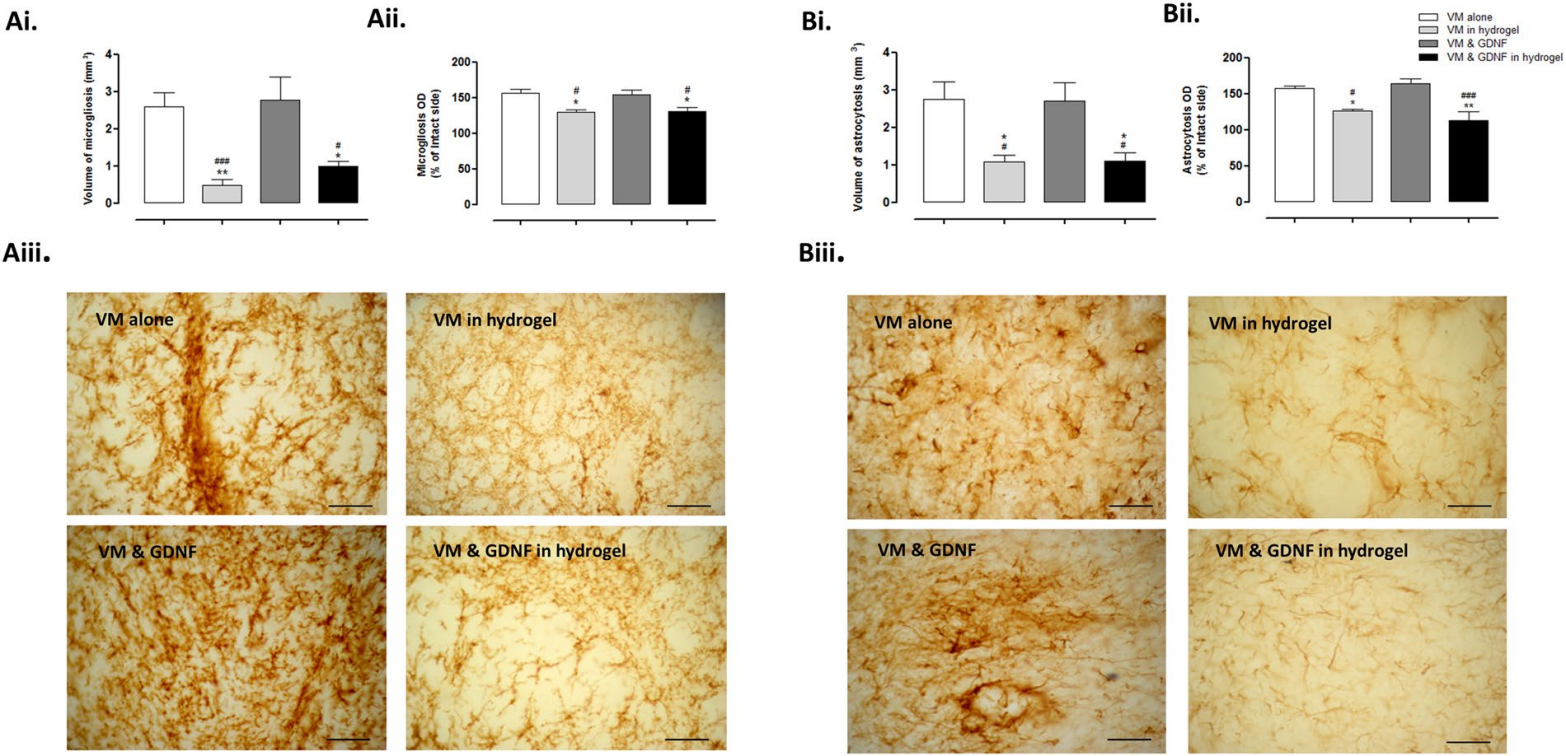
Impact of the GDNF-loaded collagen hydrogel on host immune response. The host immune response to the transplanted cells was assessed by measuring the volume and density of striatal astrocytosis and microgliosis present 12 weeks post-transplantation. IHC analysis showed that the level of both microgliosis (**A**i, ii & iii) and astrocytosis (**B**i, ii & iii) was significantly decreased by the encapsulation of VM cells in a collagen hydrogel. Importantly, this attenuation was not affected by the loading of the collagen hydrogel with GDNF. Scale bar represents 100 µm. Data are represented as mean ± SEM and were analysed by one-way ANOVA with *post-hoc* Bonferroni. ^*^ *P* < 0.05, ^**^ *P* < 0.01 vs. VM alone; ^#^ *P* < 0.05, ^###^ *P* < 0.001 vs. VM & GDNF.

In line with the results from our preliminary study (Fig. 4F,G), the VM cells elicited a host immune response in the striatal tissue surrounding the graft site (Fig. 6). However, when the VM cells were delivered in a collagen hydrogel or a GDNF-loaded collagen hydrogel there was a significant decrease in striatal microgliosis (Fig. 6A; Volume: Group, *F*_(3,30)_ = 9.792, *P* < 0.0001; Optical density: Group, *F*_(3,32)_ = 5.834, *P* < 0.01) and astrocytosis (Fig. 6B; Volume: Group, *F*_(3,28)_ = 6.659, *P* < 0.01; Optical density: *F*_(3,32)_ = 7.198, *P* < 0.01). This shows that the loading of a collagen hydrogel with GDNF does not affect its ability to act as a protective matrix to the grafted cells, reducing the host immune response.

#### Impact of the GDNF-loaded collagen hydrogel on primary dopaminergic neuron survival and striatal re-innervation

**Figure 7:**
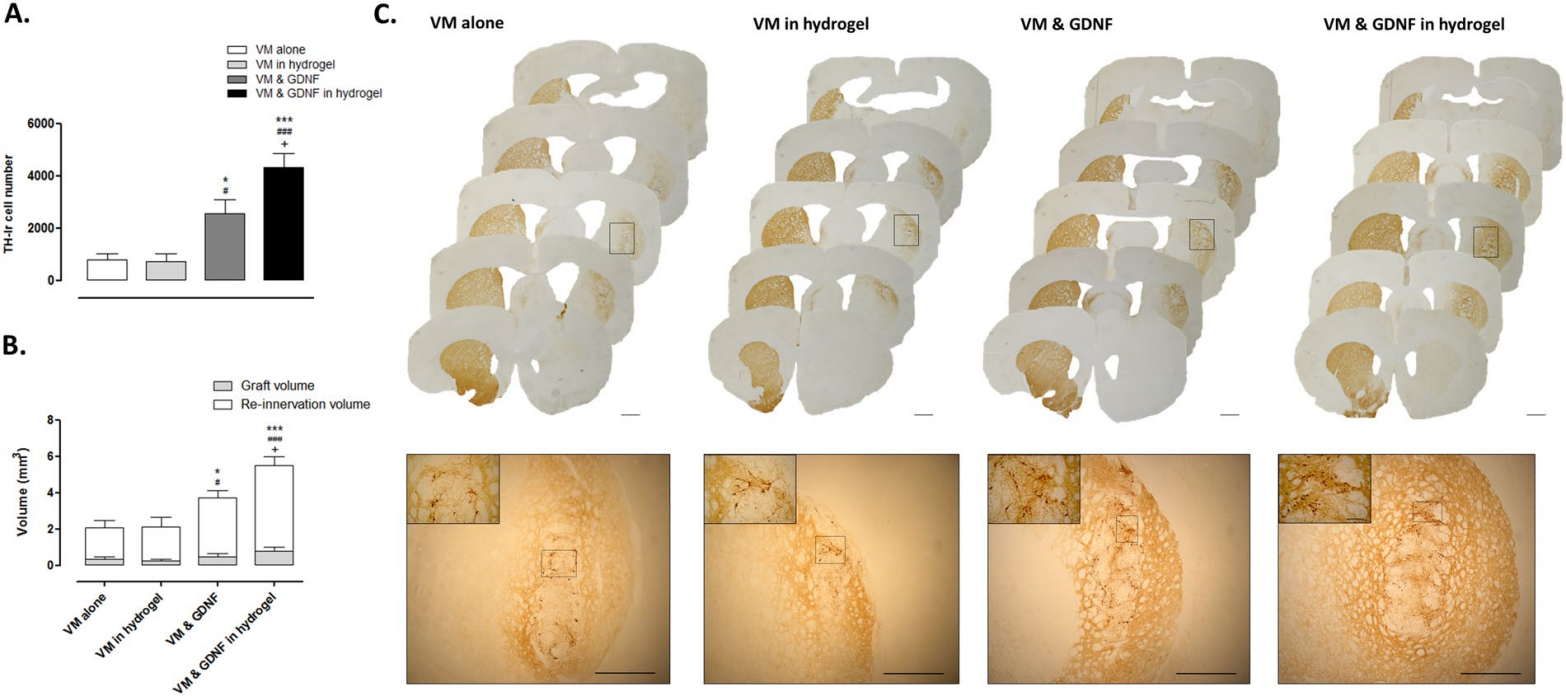
Impact of the GDNF-loaded collagen hydrogel on primary dopaminergic neuron survival and striatal re-innervation. Each animal received a unilateral transplantation of 400,000 VM cells to the lesioned striatum and both cell survival and striatal re-innervation were assessed at 12 weeks post-transplantation. TH^+^ IHC was used to identify the number of surviving dopaminergic cells throughout the striatum and their ability to re-innervate the lesioned striatum. As expected, the delivery of GDNF with cells significantly increased their survival. However, when cells were delivered in a GDNF-loaded collagen hydrogel there was a significantly greater level of cell survival (Fig. 7A,C; five-fold increase vs. VM alone). Similarly, the delivery of cells with GDNF significantly increased their striatal re-innervation. However, the delivery of cells in a GDNF-loaded collagen hydrogel resulted in a significant increase in the volume of striatal re-innervation (Fig. 7B,C; three-fold increase vs. VM alone). Scale bars represent 1 mm or 100 µm (insert). Data are represented as mean ± SEM and were analysed by one-way ANOVA with *post-hoc* Bonferroni. ^*^ *P* < 0.05, ^***^ *P* < 0.001 vs. VM alone; ^#^ *P* < 0.05, ^###^ *P* < 0.001 vs. VM in hydrogel; ^+^ *P* < 0.05 vs. VM & GDNF.

In order to evaluate the survival of transplanted grafts, the number of surviving TH^+^ dopaminergic cells throughout the striatum were counted. TH IHC identified successful transplantation of dopaminergic neurons in each group (Fig. 7A,C; Group, *F*_(3,31)_ = 15.91, *P* < 0.0001). In line with expectations, the delivery of GDNF with VM cells showed a significant increase in the number of surviving dopaminergic neurons. However, interestingly when cells were delivered encapsulated in a GDNF-loaded collagen hydrogel, there was a significant (five-fold) increase in the number of surviving cells when compared to the delivery of VM cells alone. Additionally, cell survival in a GDNF-loaded collagen hydrogel was significantly greater than that of the VM & GDNF group (1.7 fold).

We then sought to assess the ability of these surviving cells to re-innervate the striatum. Using TH IHC, the volume of striatal tissue occupied by innervation from the transplanted dopaminergic cells was measured. All VM grafts did successfully re-innervate a portion of the lesioned striatum (Fig. 7B,C; Group; *F*_(3,36)_ = 12.42, *P* < 0.0001). As expected, the volume of re-innervation was significantly increased with the delivery of GDNF with VM cells. Interestingly, in line with the five-fold increase in cell survival (above), the magnitude of striatal re-innervation was significantly greater (three-fold) from the delivery of cells alone. Additionally, striatal re-innervation from cells in the GDNF-loaded collagen hydrogel was significantly greater than that of cells delivered with GDNF alone (1.5 fold), showing that the GDNF-loaded collagen hydrogel is not only capable of increasing the number of surviving dopaminergic cells, but also the volume of innervation from these cells.

#### Impact of the GDNF-loaded collagen hydrogel on graft functionality

**Figure 8:**
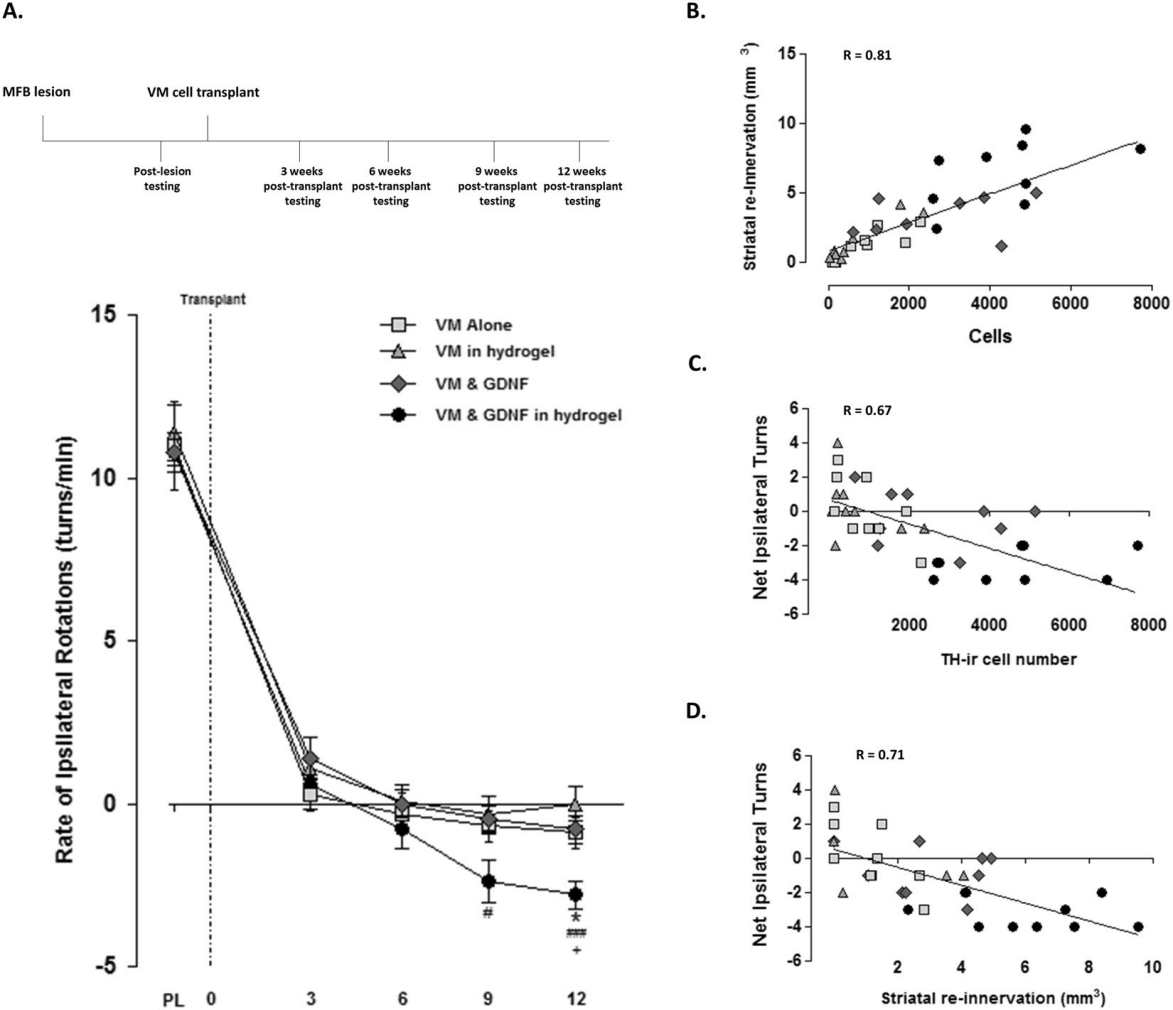
Impact of the GDNF-loaded collagen hydrogel on graft functionality. Each rat received a unilateral intra-MFB 6-OHDA lesion two weeks prior to VM transplantation. Methamphetamine induced rotations (5 mg/kg) were carried out prior to transplantation and at 3 week intervals for 12 weeks post-transplantation. Transplantation of VM grafts significantly decreased the number of ipsilateral turns made in each group. However, the delivery of cells in a GDNF-loaded collagen hydrogel resulted in a significantly greater level of functional recovery at 9 and 12 weeks post-transplantation (**A**). Given the dramatic graft survival seen with the GDNF-loaded collagen hydrogel, the relationship between cell survival, striatal re-innervation and behavioural recovery was assessed. A strong positive correlation was found between the number of surviving TH^+^ neurons and the volume of striatal re-innervation (**B**: r = 0.81). Additionally, a strong negative correlation was found between the number of net ipsilateral turns taken and the number of surviving TH^+^ cells (**C**: r = 0.67) and striatal re-innervation (**D**: r = 0.71). This indicates that the greater level of behavioural recovery seen with the GDNF-loaded collagen hydrogel is a result of the increased striatal re-innervation caused by the significant increase in TH^+^ cell survival. PL; post-lesion. Data are represented as mean ± SEM and were analysed by two-way repeated measures ANOVA with *post-hoc* Bonferroni (**A**). ^*^ *P* < 0.05 VM alone vs. VM & GDNF in hydrogel; ^###^ *P* < 0.001, ^#^ *P* < 0.05 VM in hydrogel vs. VM & GDNF in hydrogel; ^+^ *P* < 0.05 VM & GDNF vs. VM & GDNF in hydrogel.

The ability of the transplanted graft to restore function to unilaterally lesioned animals was assessed at three-weekly intervals for 12 weeks post-transplantation using methamphetamine-induced rotations. Because methamphetamine induces release of dopamine, in rats with unilateral nigrostriatal lesions, dopamine is released primarily from one side leading to rotational bias. In lesioned rats with VM transplants in the lesioned striatum, methamphetamine will also induce release of dopamine from the transplanted dopaminergic neurons thereby ameliorating the rotational asymmetry. In line with previous results, we found that the delivery of VM cell grafts significantly reduced the number of ipsilateral rotations in each group (Fig. 8A; Group x Time, *F*_(3,14)_ = 130.7, *P* < 0.0001). However, encapsulation of the VM cells in a GDNF-loaded collagen hydrogel provided a greater level of functional recovery at 9 and 12 weeks post-transplantation.

#### Correlation between VM graft survival and function

Having established that the encapsulation of VM cells in a GDNF-loaded collagen hydrogel was advantageous to their survival and outgrowth, it was important to determine whether the increased number of surviving dopaminergic neurons correlated with the observed improvement in graft function.

When we looked at the relationship between the number of surviving dopaminergic neurons and the volume of striatal re-innervation, there was a strong correlation (Fig. 8B; r = 0.81, *P* < 0.0001), indicating that the three-fold increase in striatal re-innervation was directly related to the improved cell survival caused by the encapsulation of cells in the GDNF-loaded collagen hydrogel.

The encapsulation of cells in a GDNF-loaded collagen hydrogel did not only result in an increased cell survival and re-innervation but also a greater level of behavioural recovery. This significant behavioural recovery correlates strongly with the number of surviving TH+ cells (Fig. 8C; r = 0.71, *P* < 0.0001) and also to the volume of striatal re-innervation (Fig. 8D; r = 0.67, *P* < 0.0001). This indicates that the significant reduction of net ipsilateral turns seen is directly related to the enhanced delivery and efficacy of cells in the GDNF-loaded collagen hydrogel.

#### Biodegradability of the collagen hydrogel in vivo

The absence of collagen immunohistochemical staining 12 weeks post intra-striatal delivery showed that the collagen hydrogel is biodegradable at the implanted site (not shown).

#### Expression of human-GDNF in situ

The absence of human-GDNF immunohistochemical staining 12 weeks post intra-striatal delivery showed that the transplanted GDNF was cleared from the brain prior to this 12 week time-point (not shown). This is in line with our preliminary studies (Fig. 5).

## Discussion

This present study sought to determine the impact of encapsulation in a GDNF-loaded collagen hydrogel on the survival and functional efficacy of primary dopaminergic neurons after transplantation into the Parkinsonian brain. We found that the collagen hydrogel was well tolerated in the brain, acutely retained GDNF at the injection site, and reduced the host response to the grafted cells, while also facilitating primary dopaminergic neuron survival and striatal re-innervation. Moreover, the GDNF-loaded hydrogel resulted in a 5-fold increase in primary dopaminergic neuron survival and a 3-fold increase in striatal re-innervation, which correlated with a significantly greater level of functional recovery. Given that the clinical translation of foetal-derived dopaminergic neuron regenerative therapies in Parkinson’s disease is limited by the extremely poor survival of cells post-transplantation and the consequent requirement of multiple foetal donors per transplant, if a single infusion of an injectable, non-toxic and GDNF-rich hydrogel can dramatically increase cell survival and efficacy, it can potentially reduce the number of foetal donors needed per striatal transplant. Overall this highlights the potential of growth factor enriched biomaterial matrices to enhance cell replacement therapies in neurodegenerative diseases such as Parkinson’s disease.

Cell-based therapies have emerged from a relatively simple conceptual framework as a viable therapeutic option for Parkinson’s disease: since the disease is associated with degeneration of the nigrostriatal dopaminergic neurons, then replacement of these neurons through transplantation should alleviate the disease’s motor symptoms. Over the past 30 years, a number of clinical trials have provided ‘proof-of-principle’ for this supposition, and have shown that dopaminergic neurons taken from the developing ventral mesencephalon of human foetuses can survive, integrate and function after transplantation into the adult Parkinsonian brain^8^. In spite of this, the number of patients that have benefited is small, in part due to our current dependence on VM tissue dissected from human foetuses donated after elective abortions and poor dopaminergic neuron survival after intra-cerebral transplantation. It is now well established that the majority of transplanted dopaminergic neurons die as a result of the transplantation process^12^ through matrix detachment^13^, growth factor deprivation^14^, and immune rejection^15^, all of which could be addressed using biomaterial technology.

In the present study, we used an *in situ* forming, GDNF-enriched, type 1 collagen hydrogel as a biomaterial matrix for encapsulation and transplantation of dissociated VM tissue into the striatum of Parkinsonian rats, which resulted in a dramatic increase in dopaminergic neuron survival and striatal reinnervation, with a corresponding improvement in motor function. Several features of the injectable hydrogel may be responsible for the enhanced delivery of primary dopaminergic neurons. Numerous studies suggest that the critical time-point in which 80–90% of dopaminergic neurons die, is the first 4 days post-transplantation and that it is not until after this point that dopaminergic neuron survival is stabilised^12,32,33^. Initially some apoptosis is caused by the detachment of cells from the extracellular matrix (anoikis) during tissue preparation. While many biomaterial scaffolds require chemical manipulation to improve cell adherence, collagen mimics the extracellular membrane and contains the natural Arg-Gly-Asp (RGD) tripeptide sequence that facilitates cell adhesion^34^ and thus provides an advantageous environment for cell delivery. Secondly, given that the elevated host immune response to the exogenous graft is one of the leading triggers of apoptosis post-transplantation^15^, it is of utmost importance that any delivery platform itself does not evoke an elevated host response. Collagen hydrogels, cross-linked with 4s-StarPEG have shown to be immune-neutral upon transplantation and throughout their degradation. Moreover, the delivery of cells in a GDNF-loaded hydrogel significantly decreased the host response to the transplanted graft through the formation of a physical barrier between the transplanted cells and the host neuro-immune cells. Although significant increases in cell survival were not observed with cell encapsulation alone, it is important to note that apoptosis post-transplantation is triggered by more than one factor and not solely the host immune response. While the host immune response can trigger apoptotic cell death in the established graft, the transplantation of cells into a trophic factor deprived adult striatum is associated with the vast cell death seen immediately post-transplantation^35^. Despite not finding any GDNF staining 12 weeks post-transplantation due to its relatively short half-life (3–4 days)^36^, preliminary *in vivo* studies showed that the encapsulation of GDNF in the collagen hydrogel significantly retained GDNF in the striatum immediately post-transplantation. Assuming that this was also the case when cells were encapsulated in the GDNF-loaded hydrogel, the enhanced, site-specific retention of GDNF in the striatum provided primary dopaminergic neurons with critical trophic support upon transplantation and throughout target innervation. While tackling each of these triggers alone may not be sufficient to improve graft function, the summation of enhanced cell delivery, increased trophic factor support and the attenuation of the immune response results in a significant improvement in dopaminergic cell survival, efficacy and importantly, motor function.

In summary, the present study shows, for the first time, that a growth factor-infused collagen hydrogel dramatically enhances the survival, re-innervation and functionality of primary dopaminergic neurons by reducing the host immune response to the transplanted graft and simultaneously increasing the acute retention of GDNF in the striatum. This study is therefore ‘proof-of-principle’ of the significant potential of biomaterials as a means of improving regenerative cell therapies in Parkinson’s disease, as well as other neurodegenerative diseases and therefore, warrants further investigation. Moreover, this data is in line with two recent studies of biomaterial approaches to dopaminergic neuron survival after transplantation in the brain^37,38^. In the study by Wang *et al*., 2016, a GDNF-functionalised composite poly(l-lactic acid)/xyloglucan hydrogel was shown to enhance survival of, and striatal reinnervation from, transplanted mouse VM grafts in Parkinsonian mice, while in Adil *et al*., 2017, a heparin/RGD functionalised hyaluronic acid hydrogel was shown to improve the survival of transplanted human embryonic stem cell-derived dopaminergic neurons. Taken together with the current study, this literature highlights the potential of biomaterial hydrogel scaffolds to improve the outcome of reparative cell therapies for Parkinson’s disease. Moving towards a clinical therapy, the use of bovine collagen is already approved for a variety of applications including drug delivery, wound healing, burn repair, dentistry and bone reconstruction^17,18,20,39–42^, and could therefore, be relatively easily adopted for neural applications. However, given the ethical and logistical limitations of using fetal-derived tissue for brain repair, in future studies, it will be important to determine if collagen scaffolds can also improve the outcome of stem cell-derived dopaminergic neuron transplants in the Parkinsonian brain.
